# Draft Whole-Genome Sequence of VIM-1-Producing Multidrug-Resistant *Enterobacter cloacae* EC_38VIM1

**DOI:** 10.1128/genomeA.00694-13

**Published:** 2013-09-05

**Authors:** Jennifer Villa, Esther Viedma, Joaquín R. Otero, Fernando Chaves

**Affiliations:** Servicio de Microbiología Clínica, Hospital Universitario 12 de Octubre, Madrid, Spain

## Abstract

The VIM-1-producing multidrug-resistant strain *Enterobacter cloacae* was isolated from blood culture. The strain showed multiple resistances to clinically used antibiotics, including all β-lactams, fluoroquinolones, aminoglycosides, and sulfonamides. Sequence analysis showed the presence of 14 genes associated with resistance to antibiotics, including the metallo-β-lactamase VIM-1 gene, which was located in a class 1 integron.

## GENOME ANNOUNCEMENT

*Enterobacter cloacae* is an important nosocomial pathogen and is intrinsically resistant to ampicillin and narrow-spectrum cephalosporins. Resistances to extended-spectrum cephalosporins and aztreonam are usually related to a mutational depression of the chromosomal Ambler class C β-lactamase or to the production of plasmid-mediated extended-spectrum β-lactamases (1). *E. cloacae* carbapenem-resistant clinical isolates are unusual. However, class B metallo-β-lactamases (MBLs) have been reported recently in several strains of *E. cloacae* (2, 3). MBLs confer resistance to all available β-lactams except aztreonam, and they are not inhibited by class A β-lactamase inhibitors. The most common MBLs include VIM, IMP, GIM, and SIM enzymes (4). The VIM-1 type is normally confined to *Enterobacteriaceae* isolates, and *bla*_VIM-1_ genes are located within class 1 integrons, which have been incorporated as gene cassettes. In addition, *bla*_VIM-1_ can be associated with resistance to other antibiotics, such as aminoglycosides, sulfonamides, and fluoroquinolones (5, 6).

The *E. cloacae* EC_38VIM1 strain was isolated from a blood culture of a liver transplant recipient who died as a result of septic shock secondary to cholangitis due to EC_38VIM1. The strain showed multiple resistances to clinically used antibiotics, including all β-lactams, fluoroquinolones, aminoglycosides, and sulfonamides, with the exceptions of amikacin and colistin.

Genomic DNA was extracted from an overnight culture using the DNeasy blood and tissue kit (Qiagen). Whole-genome shotgun sequencing of *E. cloacae* EC_38VIM1 was carried out by using a Roche 454 Junior sequencer according to the manufacturer’s recommended protocol to generate 28-fold coverage. *De novo* assembly was performed using Roche Newbler v2.7 (Roche), obtaining a total of 165,540,445 bp and 338,715 reads. The EC_38VIM1 assembly resulted in 90 contigs, with an *N*_50_ contig size of 262,093 nucleotides and a total length of 5,155,870 bp. Contigs were annotated using the Prokaryotic Genomes Automatic Annotation Pipeline (PGAAP) through NCBI (http://www.ncbi.nlm.nih.gov/), providing a total of 4,999 genes, 4,864 coding DNA sequence (CDS) genes, 56 pseudogenes, 4 rRNAs (5S, 16S, and 23S), and 75 tRNAs. Additionally, genome annotation was performed automatically on the Rapid Annotations using Subsystems Technology (RAST) server (7), and all open reading frames obtained from the RAST annotation were subjected to analysis using the Comprehensive Antibiotic Resistance Database (CARD) (http://arpcard.mcmaster.ca) (8). This analysis highlighted the presence of 14 genes associated with resistance to antibiotics and toxic compounds, including genes associated with specific resistance to β-lactams (AmpC β-lactamase [*bla*_MIR-8_] and metallo-β-lactamase [*bla*_VIM-1_]), aminoglycosides (streptomycin 3′-adenylyltransferase [*aadA1*] and aminoglycoside 6-adenylyltransferase [*aacA4*]), fluoroquinolones (plasmid-mediated quinolone resistance [*qnrA1*]), chloramphenicol (chloramphenicol acetyltransferase [*catB2*]), and sulfonamides (dihydrofolate reductase [*dfrB1*]). Sequencing showed that the *bla*_VIM-1_ gene was contained in a class 1 integron. The structure of the integron was *intI* (integrase gene)-*bla*_VIM-1_-*aacA4*-*dfrB1*-*aadA1*-*catB2*-*quacEΔ1/sul1* (quaternary ammonium compound resistance gene/sulfonamide resistance gene). An analysis of the genome of EC_38VIM1 identified the sequences of three plasmids, which belonged to the IncH1, IncF, and IncI1 groups. Overall, the availability of this genome sequence facilitates further comparative genomic analyses among *E. cloacae* strains with different antimicrobial susceptibility patterns in order to shed light on the classical and new antibiotic resistance mechanisms in this pathogen.

### Nucleotide sequence accession number.

The draft genome sequence of *E. cloacae* EC_38VIM1 has been included in the GenBank whole-genome shotgun (WGS) database under the accession no. ATHX00000000.
